# Reception of Dr. Marshall Hall by the Buffalo City Medical Association

**Published:** 1853-08

**Authors:** 


### EDITORIAL DEPARTMENT.

---

*Reception of Dr. Marshall Hall by the Buffalo City Medical Association.*—The occasion of Dr. Marshall Hall's visit to Niagara Falls was seized upon by the Buffalo City Medical Association, to invite him to Buffalo, and to tender to him such a tribute of respect, as his eminent talents as a physiological discoverer merited. It was very sensibly decided to have no speech-making or glorification, but to conduct the affair in such a quiet way, as would be pleasing to a sensible and modest man like Dr. Hall.

Accordingly, on the 7th of July ult., Drs. Hamilton and Strong were sent to Niagara Falls as a committee of escort; Dr. Hall having previously accepted the invitation of Dr. Wilcox, the President of the Association. On the evening of the same day the Association met at the house of Dr. Austin Flint, who introduced the distinguished guest to the President and members, with a few remarks on the services rendered by Dr. Hall to the profession, and to humanity.

It having been decided to devote the evening to medical inquiry, and to the repetition of those experiments upon which Dr. H.'s theory of nervous action was based, (the association deeming such an expression of appreciation as a higher compliment than any mere rhetoric,) Dr. Hall proceeded to perform a series of most interesting and conclusive experiments upon sundry of those scientific martyrs—speckled frogs. We are sure that our readers will not object to a brief report of Dr. Hall's experiments and remarks.

First severing the spinal marrow at its junction with the medulla oblongata, he laid the reptile upon the table to all appearance dead. Irritation of the peripheral extremities of the sensory nerves produced no muscular motion. He remarked that the animal was now laboring under the effects of shock, from which it would soon recover so far as reflex life was concerned. In a few minutes, on pinching the foot the frog withdrew it, and as it gradually recovered from the shock, it leaped vigorously about the table on the irritation of its extremities. Dr. H. then explained the philosophy of these actions. By the severance of the spinal cord all sensation and volition had been removed. In this respect the animal was dead. This he proved by the fact that so long as no irritant was applied, no motion occurred. Had sensation been present, the painful wound in its neck would induce it to move; but so long as this was the only irritant, the animal lay motionless. It was then, evident, that volition was a faculty of the brain alone. It was also evident that these were muscular motions, not dependent on the brain, but upon other parts of the nervous system. These were automatic motions.

His next proposition was, that the distribution of all sensory nerves was to the skin, and that irritation applied to other tissues would not produce muscular motion. This he proved by denuding a lower limb of its skin, and applying irritation to the muscular fibre. No motion followed. Upon the opposite limb, however, any irritant was followed by muscular motion. He then cut down upon the femoral nerve of that limb, and severed it. This also destroyed muscular motion. By these experiments he proved that a circuit existed, and that this circuit extended above the point of the division of the nerve.

First, he had proved that the point of departure of nervous influence, or what we call the origin of the nerves, was in the skin. That an excitant being applied to the peripheral or skin extremity of a sensory nerve, an influence was propagated along its trunk to the spinal marrow — that some change or action there, reflected an influence along a nerve returning to the point of excitation — that this nerve produced muscular motion, and finally, that the concurrence of the brain was unnecessary to these automatic motions.

Automatic motions consisted first of those of prehension, which he illustrated by the well known fact that a sleeping infant closes its hand on any object placed within its palm — second, of motions concerned in deglutition and expulsion of the contents of viscera; and lastly, of the motions under the control of the ganglionic system, such as respiration and circulation.

Finally, that the ganglionic nerves were all nerves of motion, and that no sensation is ever felt in organs supplied by them, unless from diseased action, pain being the only feeling they possess. That the ganglionic system is separated from the excito-motor system, and that the latter is not dependent on the former for any power, he proved by removing all the thoracic and abdominal viscera, and demonstrating that the motions of the muscles were unaffected by their withdrawal.

Probably many of our readers are familiar with these experiments and their deductions; but we assure them, that it was no small pleasure to see them under the hands of him who first demonstrated their important application. Any one can appreciate the fact, that a discoverer is ever more lucid in his explanation of his idea, than second-hand delineators of them.

Among the most attentive observers and listeners of Dr. Hall, was Ex-President Fillmore, (Chancellor of the University of Buffalo,) whose presence that evening was itself a striking compliment to Dr. Hall, and we may venture to add, an evidence of his own appreciation of the value of medical research.

So pleasant and instructive an evening could not pass without some festivity. Accordingly the Association and guests adjourned to the supper room, whence after a cheerful hour they dispersed. Dr. Hall remained in town for a day or two, receiving, with his family, the hospitalities of Drs. White and Hamilton. S. B. H.

